# Elicitation of protective immune responses using a bivalent H5N1 VLP vaccine

**DOI:** 10.1186/1743-422X-5-131

**Published:** 2008-10-28

**Authors:** Corey J Crevar, Ted M Ross

**Affiliations:** 1 Center for Vaccine Research, University of Pittsburgh, Pittsburgh, PA, USA; 2 Department of Microbiology and Molecular Genetics, University of Pittsburgh, Pittsburgh, PA, USA

## Abstract

**Background:**

Currently licensed human vaccines are subtype-specific and do not protect against pandemic H5N1 viruses. Previously, our group has reported on the construction of an influenza virus-like particle (VLP) as a new generation candidate vaccine. A mixture of influenza H5N1 VLPs representing clade 1 and 2 viruses were examined for the ability to elicit protective immunity against isolates from various clades and subclades of H5N1.

**Results:**

Mice were vaccinated intramuscularly with each VLP individually, the mixture of VLPs, a mixture of purified recombinant hemagglutinin (rHA), or mock vaccinated. Elicited antibodies were assayed for the hemagglutination-inhibition (HAI) activity against clades 1 and clade 2 isolates. Mice vaccinated with each VLP individually or in a mixture had robust HAI responses against homologous viruses and HAI responses against the clade 2.3 virus, Anh/05. However, these vaccines did not induce an HAI response against the clade 2.2 virus, WS/05. Interestingly, clade 2 VLP vaccinated mice were protected against both clade 1 and 2 H5/PR8 viruses, but clade 1 VLP vaccinated mice were only protected against the clade 1 virus. Mice vaccinated with a mixture of VLPs were protected against both clade 1 and 2 viruses. In contrast, mice vaccinated with a mixture of rHA survived challenge, but lost ~15% of original weight by days 5–7 post-challenge.

**Conclusion:**

These results demonstrate that a multivalent influenza VLP vaccine representing different genetic clades is a promising strategy to elicit protective immunity against isolates from emerging clades and subclades of H5N1.

## Introduction

Since re-emerging in 2003, avian influenza viruses of the H5N1 subtype have spread from Southeast Asia across central Asia and the Middle East into Europe and Africa by infecting wild birds and poultry. New influenza viruses and genotypes are emerging each year and are leading to significant genetic variation among H5N1 viruses [1]. Currently, 10 clades of H5N1 isolates have been identified in birds. Recent human isolates have clustered into two distinct clades, clade 1 and clade 2, with clade 2 further being divided into subclades 2.1, 2.2, and 2.3. Although H5N1 remains an avian virus, not yet adapted to efficient transmission between humans, there is concern that small genetic changes may significantly alter the pandemic potential of this virus, allowing it to emerge as the next influenza pandemic strain. Therefore, a potential vaccine against H5N1 influenza isolates should ideally protect against the diverse set of currently circulating strains and future H5N1 variants.

One of the challenges faced by influenza vaccine developers is the ability to protect populations in the face of emerging and spreading pandemics. The next influenza pandemic may be caused by an H5N1 virus and if so, it is not known which clade or subclade may be responsible. Therefore, vaccine(s) that elicit broadly-reactive immune responses against viruses from multiple or all H5N1 clades are critical targets for vaccine manufacturers. Previously, our group described the development and immunogenicity elicited by a recombinant H5N1 influenza virus-like particle (VLP) vaccine in mice and ferret models [2-4]. This VLP vaccine does not require the use of any live influenza virus in the manufacturing process that would significantly complicate the safety and process of mass production. VLP-based vaccines are a promising, innovative technology for safe and efficacious vaccines against many viral diseases [5-10], including influenza viruses [4]. VLP vaccines are particularly advantageous to meet future global pandemics because these vaccines 1) need short lead times for development of "new-to-the-world" vaccines, 2) use recombinant DNA technology to facilitate rapid strain matching, 3) provide the correct three-dimensional antigenic conformation of the HA and NA for "native-like" presentation of antigens to the immune system, and 4) show promise in being able to induce a robust and broadly reactive immunity against drifted virus variants at low doses without the addition of an adjuvant [2-4,11].

Conventional seasonal influenza vaccines use a trivalent mixture of split viruses, containing two influenza A subtypes (H1N1 and H3N2) and one variant of influenza B virus without the loss of immunogenicity to an individual subtype within the vaccine formulation. Therefore, we speculated that mixing influenza H5N1 VLPs could be a promising strategy to elicit protective immunity against various clades and subclades of H5N1. A multivalent pandemic influenza VLP vaccine has not been investigated despite the need to evaluate alternative influenza vaccine strategies that elicit immune responses against viral isolates from different clades. In this study, two H5N1 VLPs representing clade 1 and clade 2 isolates were mixed together to generate a bivalent vaccine formulation. The mixed VLP vaccine was administered to mice and the protective immune responses were compared to each individual VLP vaccine, rHA, and a mock control.

## Results

### Induction of antibodies following VLP immunizations

**Figure 1:**
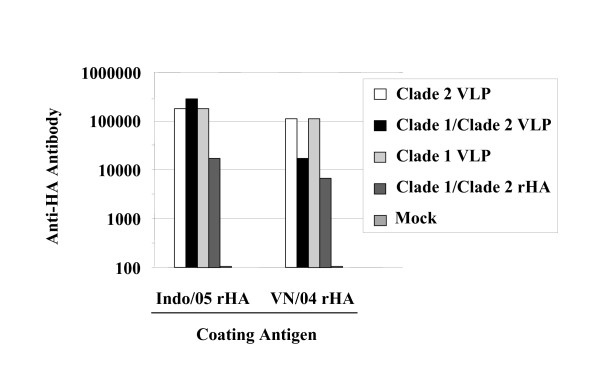
**Antibodies elicited by vaccination**. Mice (n = 8) were vaccinated via intramuscular injection at weeks 0 and 3 with VLPs (clade 1 or clade 2) or recombinant HA, or PBS only (Mock). At week 5, serum from each group was pooled and tested for anti-HA antibodies by ELISA. The endpoint dilution titer was determined against Indo/05 rHA or VN/04 rHA. Pre-immune sera from mice had no detectable specific anti-HA antibodies.

Previously, our group has demonstrated the effectiveness of influenza virus-like particles to elicit immune responses against HA, NA, and M1 from clade 1 and clade 2 H5N1 isolates [2]. In this study, clade 1 and clade 2 H5N1 VLPs were formulated in a mixture prior to administration to mice to determine if there was a loss of immunogenicity compared to each VLP administered individually. Recombinant baculoviruses expressed individual HA, NA, or M1 proteins from A/Viet Nam/1203/2004 (clade 1) or the A/Indonesia/05/2005 (clade 2) viruses. These proteins assembled into viral particles, were efficiently secreted into the supernatant, and were purified, as previously described [2-4]. Mice (BALB/c; n = 8) were vaccinated (week 0 and 3) via intramuscular injection with each individual influenza VLP or VLPs were mixed in an equal ratio (1:1) based upon HA content. Purified rHAs were used as a positive control. At week 5, mice vaccinated with VLPs individually or in a mixture had similar serum IgG endpoint dilution titers (~1:10^5^) to the clade 2 rHA (Figure. 1). In contrast, mice vaccinated with the mixture of VLPs had a log lower anti-HA titer (1:10^4^) to the clade 1 rHA compared to mice administered each VLP individually (1:10^5^). IgG_2a _was the dominate isotype (data not shown).

**Figure 2:**
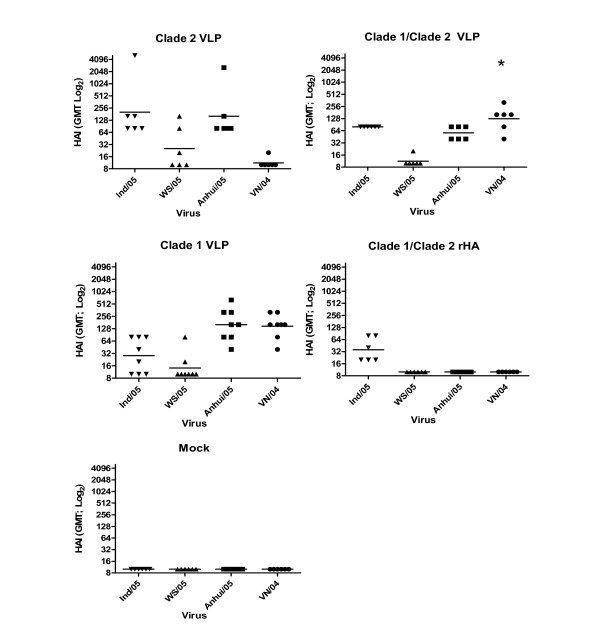
**Hemaggutination-inhibition (HAI) titers**. Week 5 serum HAI antibody responses were assessed against H5N1 clade 1 (VN/04) and clade 2 (Indo/05, WS/05, Anh/05) viruses. Bars indicate geometric mean titer (GMT) +/- 95% CI. A) Clade 2 VLP; B) Clade 1/Clade 2 VLPs; C) Clade 1 VLP; D) Clade 1/Clade 2 rHA; E) Mock. * indicates p < 0.05 compared to Clade 1 VLPs.

Serum samples were evaluated for the ability to prevent virus-induced agglutination of horse RBCs. At week 5, 100% of the mice vaccinated with clade 2 VLP (201 +/- 834 GMT) to the homologous clade 2 Indo/05 virus (Figure. 2A and 2C). Mice with an HAI value ≥ 1:40 were considered positive. One clade 2 VLP vaccinated mouse consistently had higher HAI titers, regardless of the virus tested (Figure. 2A). Mice vaccinated with clade 2 VLPs did not have any cross-reactive HAI antibodies to VN/04 (Figure. 2A), however, all mice vaccinated with 1/clade 2 VLPs had HAI titers against VN/04 (Figure. 2B). All the mice vaccinated with the clade 1 VLP had HAI activity against the clade 1 VN/04 virus and 50% of the clade 1 VLP vaccinated mice had HAI antibody titers against the clade 2.1 Indo/05 virus (Figure. 2C). As predicted, all mice vaccinated with the mixture of clade 1/clade 2 VLPs elicited HAI antibodies against the both VN/04 virus (115 +/- 36 GMT) and Indo/05 virus (80 +/- 0 GMT) (Figure. 2B). Only 33% of the mice vaccinated with a mixture of clade 1/clade 2 rHA had an HAI titer (36 +/- 12 GMT) against the Indo/05 virus (Figure. 2D).

VLP elicited antibodies were also tested against viruses from other clade 2 subclades. All the mice vaccinated with the clade 2 VLPs had HAI activity against the clade 2.3 Anhui virus (Figure 2A), however there was lower HAI activity against the clade 2.2 WS/05 (Figure. 2A) or BGH/05 (data not shown) viruses. All mice vaccinated with the clade 1 VLPs had HAI titers against Anh/05 and only 1 of the 8 mice (12.5%) had HAI activity against WS/05 (Figure. 2C). Similar results were observed in mice vaccinated with the mixture of clade 1/clade 2 VLPs.

### Influenza virus challenge

**Figure 3:**
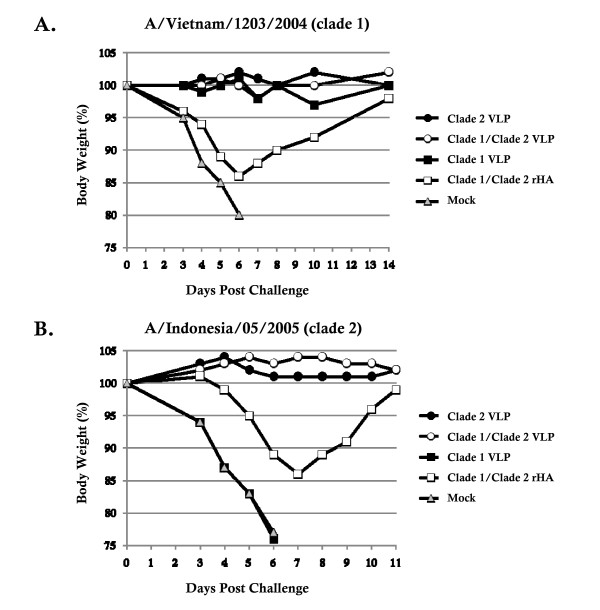
**Influenza virus challenge**. At week 5, mock vaccinated mice or mice vaccinated with vaccines were challenged intranasally with reassortant H5/PR8 influenza viruses representing (A) VN/04 or (B) Indo/05. Mice were monitored daily for weight loss, activity, and survival. Body weight is plotted as percentage of the average initial weight. Mice that lost greater than 30% body weight were sacrificed.

Mice were challenged with H5/PR8 reassortant viruses representing either the clade 1 VN/04 virus or the clade 2 Indo/05 virus. Mice were observed for clinical signs of infection (ruffled fur, dyspenea, lethargy) and weight loss. Unvaccinated mice that were challenged with either virus lost ≥ 20% of original body weight by day 6 post-infection (Figure. 3). Mice vaccinated with the clade 1, clade 2, or a mixture of clade 1/clade 2 VLPs and then challenged with the clade 1 VN/04 virus had no weight loss (Figure. 3A) and had no clinical signs of infection over the period of observation (Table 1). Mice vaccinated with the mixture of rHA proteins lost ~15% of their original body weight by day 6 and then began to recover.

**Table 1:** Virological and pathological assessment following Indo/05 challenge

*Vaccine*^*a*^	*% Body Weight*^*b*^	*% Body Weight*^*c*^	*Plaque Titer*^*d*^	*Activity*^*e*^	*Dyspnea*^*f*^
Clade 2 VLP	102%	103%	<1.00e+2	0	0
Clade 1/Clade 2 VLP	102%	101%	5.25e+3	0	0
Clade 1 VLP	95%	76%	5.73e+6	2	1
Clade 1/Clade 2 rHA	98%	89%	3.54e+5	1	0
Mock	94%	77%	5.94e+6	2	1

^*a*^Vaccine administered at weeks 0 and 3.

^*b*^Percentage of original weight at day 3 post-challenge.

^*c*^Percentage of original weight at day 6 post-challenge.

^*d*^Particle forming units (pfu) per milliliter (ml) in the lungs of mice at day 3 post-challenge.

< 1.00e + 2 = Viral titers less than 100 pfu/ml.

^*e *^Activity score. 0 = Full activity, 1 = slow to respond to touch, but still mobile, 2 = little response to touch.

^*f*^Dyspnea. 0 = no shortness of breadth. 1 = heavy breathing and shortness of breadth.

In contrast to clade 1 virus challenge, mice vaccinated with clade 1 VLPs and then challenged with the clade 2 Indo/05 virus were not protected from challenge (Figure. 3B). All these mice showed signs of disease (Table 1), lost ≥ 20% of their original weight, and died by day 6 post-challenge. Mice vaccinated with clade 2 VLPs or the mixture of clade 1/clade 2 VLPs were all protected from clade 2 Indo virus challenge. Similar to clade 1 virus challenge, mice vaccinated with a mixture of clade 1/clade 2 rHA proteins lost ~15% of their body weight, but the weight loss was more gradual with the peak weight loss at day 7. These mice recovered more quickly than mice challenged with clade 1 virus.

On day 3 post-challenge with Indo/05, the lungs of 5 mice from each group were collected and tittered for virus (Table 1). Mice vaccinated with the clade 2 VLPs or a mixture of clade 1/clade 2 VLPs had low viral titers following challenge with the clade 2 Indo virus. Whereas, mice vaccinated with clade 1 VLPs or a mixture of clade 1/clade 2 rHA proteins had viral titers similar to unvaccinated mice (Table 1).

## Discussion

A new influenza pandemic is inevitable. The culprit could be an avian influenza strain from the H5N1 subtype. To date, many vaccine candidates have been developed against avian H5N1 influenza viruses, some traditional and some novel. It is clear that to respond to an emerging threat posed by an avian influenza outbreak, a vaccine must be immunogenic and be able to induce immunity, against potential viral drift variants. In addition, recombinant-based vaccine options need to permit the ability to quickly adapt a vaccine to match the circulating strain of virus and then manufacture the relevant vaccine in a short period of time. Alternatively, a vaccine must elicit a broadly reactive immune response that neutralizes viruses from various clades of H5N1 influenza.

In this study, we investigated the ability of a vaccine representing isolates from clade 1 and clade 2 of the H5N1 subtype of influenza to elicit protective antibodies against viruses from both clades. The clade 1 and clade 2 VLPs were immunogenic in mice and protected against virus challenge, regardless if the VLP vaccines were administered individually or in a bivalent formulation. The clade 2 VLP elicited a high degree of cross-protection than the clade 1 VLP (Figure. 3).

The cross-reactivity of the single-strain vaccines is more limited than that of the mixed vaccine. A mixture of these VLPs elicited HAI antibodies that inhibited the agglutination of horse RBCs by both the clade 1 and clade 2 isolates that were homologous to the vaccines (Table 1). These titers were statistically similar to the titers elicited by each VLP vaccine alone. As expected, there were no cross-clade reactive HAI antibodies elicited by each VLP vaccine administered individually. Antibodies elicited by clade 1 or clade 2 viruses have limited cross-reactive HAI activity. Multivalent spilt vaccines consisting of an H1N1, H3N2 and B are commonly administered each year to people for seasonal influenza viruses [12]. The results presented in this report indicate that a bivalent or multivalent vaccine based upon emerging strains with high pandemic potential should be considered for further evaluation in clinical trials. A low dose, multivalent vaccine, without an adjuvant, may be appropriate for stock-piling or for pre-pandemic immunization of high risk individuals.

In order to examine the breadth of HAI activity against clade 2 isolates from different subclades, antisera was tested against viral isolates representing clades 2.2 and 2.3. The clade 2 VLP vaccine was derived from a clade 2.1 isolate and we have previously demonstrated no cross-reactive HAI activity to clade 2.2 viruses, but elicited antibodies do show some level of HAI cross-reactivity to some clade 2.3 isolates (personal observations). We chose to examine the cross-reactivity to viruses within clade 2, and not clade 1 or 3, since the most recent reports indicate that isolates from the clade 2 lineage are spreading to Europe, Middle East and Africa. Mixing clade 1 and clade 2 VLPs did not increase HAI activity against the clade 2.2 isolate WS/05 (Table 1) or BGH/05 (data not shown). Interestingly, these antibodies maintained HAI activity to the clade 2.3 virus (Anh/05), however, the average HAI GMT titer was lower than the HAI titer elicited by the clade 2 VLP alone (Figure. 2). These results are highly significant and demonstrate that a multivalent vaccine against H5N1 appears to be a plausible strategy to combat the diversity of clades and subclades of H5N1 influenza. Future studies will need to determine the efficacy and breadth of immunity elicited by a multivalent vaccine composed of VLPs representing various clades or subclades of H5N1.

There was a direct correlation to survival of mice to lethal challenge and the level of HAI activity elicited by the vaccines. Recent studies in ferrets have shown that HAI antibody titers raised against vaccine candidates from avian H5N1 influenza viruses do not always correlate with protection against a lethal challenge virus [3,11,13-15]. These differences in protection against lethal challenge between mice and ferret may be due to the differences in the anatomy of these two species and the site of viral replication. Influenza replicates in the nasal and upper respiratory tract in ferrets, however, the bolus of virus delivered to the mouse is delivered into the lower respiratory tract thereby resulting in different pathogenicity.

The isotype of the antibody may play as an important a role in protection as the antibody titer. Whether mixed together or individually, these VLPs elicited an IgG_2a _antibody profile (data not shown). Previous studies from our group in mice indicated that these VLPs elicited high titer serum IgG_2a _following intramuscular injection [2,3]. The IgG_2a _isotype has been associated with protection against influenza, even in the absence of neutralizing antibodies [16] and appears pivotal in anti-viral immunity [17]. Intramuscular vaccination of rHA elicited a mixed IgG_1_/IgG_2a _isotype response. IgG_2a _correlates with increased viral clearance and enhanced protection against challenge, whereas IgG_1 _secretion is more often associated with binding ELISA assays and microneutralization assays [16]. IgG_2a _is the most effective isotype at fixing complement [18] and binding to Fc receptors on macrophages [19,20] and NK cells [20]. Enhanced antibody uptake by these cells increases opsonization and antibody-dependent mediated cytotoxicity (ADCC) [21], as well as clearance of influenza in the respiratory mucosa [22]. Therefore, one of the advantages of these VLP vaccines may be enhanced viral clearance, independent of HAI or neutralization activity.

In addition to humoral responses, VLP vaccines have been shown to elicit cellular immunity [2] and therefore, we cannot rule out that cellular immune responses elicited by these vaccines played a role in the protection. Protection against influenza infection is a multifactorial phenomenon, with both innate and cellular responses (NK, NKT, and CD8+ T cell responses) associated with clearance of influenza viral infected cells [23]. The role of innate and adaptive cellular responses to influenza vaccination has not been extensively studied in humans. Even fewer studies have addressed the role of vaccine-induced cellular responses to influenza virus on the outcome of infection, particularly in neonates and the elderly. We recently showed that these VLP vaccines could elicit T cell specific HA and M1 responses [2,3] and reduced the induction of pro-inflammatory cytokines and granulocytes into the lungs following either intramuscular or intranasal VLP vaccination.

The recombinant influenza virus-like particle vaccines preserve native, conformational antigenic epitopes of influenza proteins in the context of a highly immunogenic, non-infectious structure. VLPs elicit immune responses and protection at low doses (HA content) and without the use of an exogenous adjuvant [2], both of which potentially reduce reactogenicity of the vaccine. Recombinant VLP vaccines avoid the potential safety risks associated with live attenuated or whole virus pandemic influenza vaccines, because the manufacturing process does not require infectious virus.

## Conclusion

Each season, manufactures generate seasonal influenza vaccines based on a mixture of three viruses representing a H1N1, H3N2, and a B influenza virus. Therefore, we speculated that a multivalent vaccine representing strains from different clades of H5N1 influenza could elicit protective immunity. Two H5N1 virus-like particle vaccines representing clade 1 and clade 2 isolates were mixed together to generate a bivalent vaccine formulation. Mice vaccinated with each VLP individually or in a mixture had robust HAI responses. Mice vaccinated with a mixture of VLPs were protected against both homologous clade 1 and 2 viruses and the heterologous clade 2.3 Anh/05 virus. However, the vaccine did not elicit HAI activity against the clade 2.2. These results demonstrate that mixing vaccines from different clades or subclades can broaden the immune response against H5N1 isolates. This approach is promising strategy and with additional vaccines representing additional clades/subclades could be used to generate a mutilvalent H5N1 VLP vaccine.

## Methods

### Viruses and nomenclature

H5N1 influenza type A reassortant viruses (see below) virus isolates were used in this study. Abbreviations for the H5N1 viral isolates were Clade 1: A/Viet Nam/1203/2004 (VN/04); Clade 2.1: A/Indonesia/05/2005 (Indo/05); Clade 2.2: A/Bar headed goose/Qinghai/1A/2005 (BHG/05), A/Whooper swan/Mongolia/244/2005 (WS/05); Clade 2.3: A/Anhui/1/2005 (Anh/05).

### Cloning of HA, NA, and M1 genes and the generation of recombinant baculoviruses

Development of these virus-like particles have been previously described [2]. Briefly, the HA, NA, and M1 genes coding for the proteins contained in each H5N1 VLP vaccine were synthesized by GeneArt (Germany) based upon sequences submitted to the Influenza Sequence Database (July 29, 2005), followed by cloning and expression from recombinant bacmids infected into *Spodoptera frugiperda *Sf9 insect cells (ATCC CRL-1711) [3,4]. At 72 hours post-transfection, VLPs were harvested and purified using sucrose gradient ultracentrifugation and ion-exchange chromatography. Virus-like particle formation was confirmed by Western blot (data not shown) as described by Pushko *et al*. [4]. Dose was measured by hemagglutinin content using quantitative single radial immunodiffusion (SRID) as described by Wood *et al*. [12]. Briefly, hemagglutinins were purified from Sf9 insect cells (A/Viet Nam/1203/2004 and A/Indonesida/05/2005, Lot #083006) and injected into sheep to raise specific hyperimmune antiserum. Reagents (CBER Ref. #45-0503RA-2) from the U.S. Center for Biologics Evaluation and Research (CBER) were utilized simultaneously. VLPs were diluted and allowed to diffuse overnight in 1% agarose containing the pre-determined optimal dilution of anti-HA sheep reference sera. The agarose gel was stained with Coomassie Blue and the diameter (mm) of antigen-antibody precipitation rings was measured with a micro-comparator.

### Animals and vaccinations

BALB/c mice (*Mus musculis*, females, 6–8 weeks) were purchased from Harlan Sprague Dawley, (Indianapolis, IN, USA). Mice were housed in microisolator units and allowed free access to food and water and were cared for under USDA guidelines for laboratory animals. Mice (8 mice per group) were vaccinated with rHA (600 ng) or purified VLPs (600 ng), based upon HA content, via intramuscular injection at week 0 and then boosted with the same dose at week 3. Blood was collected from anesthetized mice via the orbit and transferred to a microfuge tube. Tubes were centrifuged and sera was removed and frozen at -80 ± 5°C. All procedures were in accordance with the NRC Guide for the Care and Use of Laboratory Animals, the Animal Welfare Act, and the CDC/NIH Biosafety in Microbiological and Biomedical Laboratories.

### Enzyme-linked Immunoabsorbant Assay

A quantitative ELISA was performed to assess anti-HA specific IgG in immune serum. Purified rHA (30 ng) was used to coat each well of a 96 well plate as previously described [3,11,13,14]. Plates were blocked (25°C for 2 hr) with PBS containing Tween 20 (0.05%) and nonfat dry milk (5%) and then incubated with serial dilutions of each serum sample (25°C for 2 hr). Following thorough washing in PBS-Tween 20 (0.05%), samples were incubated (25°C for 1 hr) with biotinylated goat anti-ferret IgG (1:5000) diluted in PBS-Tween 20 (0.05%) and nonfat dry milk (5%). The unbound antibody was removed, and the wells were washed. Strepavidin-HRP (1:7000) was diluted in PBS-Tween 20 (0.05%) and incubated (25°C for 1 hr). Samples were incubated with TMB substrate (1 hr), and the colorimetric change was measured as the optical density (O.D., 405 nm) by a spectrophotometer (Dynex Technologies, Chantilly, VA, USA). The O.D. value of the age-matched naïve sera was subtracted from the samples using antisera from vaccinated mice. Results were recorded as the geometric mean titer (GMT) ± the standard error of the mean (SEM).

### Hemagglutination-inhibition (HAI) assay

Hemagglutination inhibition (HAI) assays were conducted essentially as previously described [2]. To inactivate non-specific inhibitors, aliquots of each serum sample were separately treated with receptor destroying enzyme (RDE) prior to being tested with a final serum dilution of 1:10 (starting dilution for the assays). Samples were serially diluted 2-fold into V-bottom 96 well microtiter plates. An equal volume of H5N1 reassortant viruses, adjusted to approximately 8 HA units/50 μl was added to each well. The plates were covered and incubated at room temperature for 30 minutes followed by the addition of freshly prepared 1% horse erythrocytes (hRBCs) (Lampire Biologicals, Pipersville, PA, USA) in PBS. The plates were mixed by agitation, covered, and allowed to set for 60 minutes at 25°C. The HAI titer was determined by the reciprocal of the last dilution which contained non-agglutinated hRBCs. Positive and negative serum controls were included on each plate. Geometric mean HAI titers and standard error were calculated for each group.

### Propagation of H5N1 reassortant viruses

The H5 HA and N1 NA of the reassortant H5N1 (H5/PR8) viruses were derived from influenza A/VN/1203/2004 (VNH5N1-PR8/CDC-RG; termed VN/04) and A/Indonesia/05/2005 (Indo/05/2005(H5N1)/PR8-IBCDC-RG2; termed Indo/05) viruses and the internal protein genes was derived from the A/Puerto Rico/8/1934 (PR8) donor virus (kindly provided by Ruben Donis, Influenza Division, Centers for Disease Control and Prevention, Atlanta, GA, USA). Each virus requires the addition of 0.5 μg/ml TPCK-treated typsin to induce plaques in minimal essential medium (MEM) containing 0.8% agarose on chick embryo fibroblasts (CEF) or MDCK cells, as determined by Ruben Donis at the CDC. These reassortant viruses administered intranasally are not pathogenic to chickens (Ruben Donis, CDC, personal communication) or ferrets (personal observation). Lethal doses of each virus (Indo/05; 1.8 × 10+5 pfu/ml and VN/04; 1.6 × 10+4 pfu/ml) were administered to 8-week old mice as previously described [2].

Viral stocks of each reassortant virus were propagated in the allantoic cavity of 9- to 11-day-old embryonated specific pathogen-free (SPF) hen's eggs at 37°C. The allantoic fluids from eggs inoculated with each virus was harvested 24 h post-inoculation and tested for hemagglutinating activity. Eggs inoculated with reassortant viruses were incubated at 33°C and were harvested 3 days post-inoculation. Infectious allantoic fluids were pooled, divided into aliquots, and stored at -80°C until used for studies. The 50% tissue culture infectious dose (TCID_50_) for each virus was determined by serial titration of virus in Madin-Darby canine kidney (MDCK) cells and calculated by the method developed by Reed and Muench [24]. All experiments, including animal studies with infectious reassortant viruses, were conducted using enhanced BSL-2 containment procedures in laboratories approved for use by the USDA and Centers for Disease Control and Prevention. Animal experiments were approved by the National Institutes of Health Animal Care and Use Committee.

### Plaque Assay with and without Trypsin

MDCK cells plated in 6-well tissue culture plates were inoculated with 0.1 ml of virus serially diluted in Dubecco's modified Eagle's medium (DMEM). Virus was adsorbed to cells for 1 h, with shaking every 15 min. Wells were overlaid with 1.6% w/v Bacto agar (DIFCO, BD Diagnostic Systems, Palo Alto, CA, USA) mixed 1:1 with L-15 media (Cambrex, East Rutherford, NJ, USA) containing antibiotics and fungizone, with or without 0.6 μg/ml trypsin (Sigma, St. Louis, MO, USA). Plates were inverted and incubated for 2–3 days. Wells were then overlaid with 1.8% w/v Bacto agar mixed 1:1 with 2× Medium 199 containing 0.05 mg/ml neutral red, and plates were incubated for two additional days to visualize plaques. Plaques were counted and compared to uninfected cells.

### Protection from lethal viral challenge

Vaccinated mice were challenged with a lethal dose (10 LD_50_) of one of the two H5N1 reassortant viruses as previously described [2]. Mice were monitored daily for clinical signs of influenza infection and body weight was recorded each day. Mice that lost greater than 25% of body weight were euthanized. The ability of each vaccine to protect against homologous or heterologous challenge was compared to separate groups of naive, unvaccinated control mice that were challenged with each reassortant virus.

Lung virus titers were determined using a plaque assay [2,3]. Briefly, lungs from mice infected with virus were collected and single cell suspensions via passage through a 70 mM mesh (BD Falcon, Bedford, MA, USA) in 4 ml of PBS. Cell suspensions were frozen (-80C) for 1 h, and then thawed, centrifuged at 1000 × g for 10 min, and then the supernatants were collected and stored at -80C. Madin-Darby Canine Kidney (MDCK) cells were plated (5 × 10e+5) in each well of a six-well plate. Virus was diluted (1:100 to 1:1000) and overlayed onto the cells in 100 ul of DMEM supplemented with penicillin-streptomycin and incubated for 1 hr. Virus-containing medium was removed and replaced with 2 ml of L-15 medium plus 0.8% agarose (Cambrex, East Rutherford, NJ, USA) and incubated for 48 hrs at 37C with 5% CO_2_. Agarose was removed and discarded. Cells were fixed with 70% EtOH, and then stained with 1% crystal violet for 15 min. Following thorough washing in dH2O to remove excess crystal violet, plates were allowed to dry, plaques counted, and the plaque forming units (pfu)/ml were calculated.

### Statistical analysis

Statistical analyses were performed using a two-tailed *t*-test with equal variance. Samples from VLP-vaccinated animals were compared to unvaccinated animals and significance was considered at a *p*-value ≤ <0.05.

## Competing interests

The authors declare that they have no competing interests.

## Authors' contributions

CJC provided substantial input to study design, executed the experiments, and analyzed data. TMR designed and analyzed data and wrote the manuscript. All authors read and approved the final manuscript.
